# Homologous overexpression of *rfaH* in *E. coli* K4 improves the production of chondroitin-like capsular polysaccharide

**DOI:** 10.1186/1475-2859-12-46

**Published:** 2013-05-09

**Authors:** Donatella Cimini, Mario De Rosa, Elisabetta Carlino, Alessandro Ruggiero, Chiara Schiraldi

**Affiliations:** 1 Department of Experimental Medicine, Section of Biotechnology and Molecular Biology, Second University of Naples, via de Crecchio 7, Naples, 80138, Italy

**Keywords:** *E. coli* K4, Capsular polysaccharide, *rfaH*, Antiterminator, Chondroitin sulfate, UDP-precursors

## Abstract

**Background:**

Glycosaminoglycans, such as hyaluronic acid, heparin, and chondroitin sulfate, are among the top ranked products in industrial biotechnology for biomedical applications, with a growing world market of billion dollars per year. Recently a remarkable progress has been made in the development of tailor-made strains as sources for the manufacturing of such products. The genetic modification of *E. coli* K4, a natural producer of chondroitin sulfate precursor, is challenging considering the lack of detailed information on its genome, as well as its mobilome. Chondroitin sulfate is currently used as nutraceutical for the treatment of osteoarthritis, and several new therapeutic applications, spanning from the development of skin substitutes to live attenuated vaccines, are under evaluation.

**Results:**

*E. coli* K4 was used as host for the overexpression of RfaH, a positive regulator that controls expression of the polysaccharide biosynthesis genes and other genes necessary for the virulence of *E. coli* K4. Various engineering strategies were compared to investigate different types of expression systems (plasmid vs integrative cassettes) and integration sites (genome vs endogenous mobile element). All strains analysed in shake flasks on different media showed a capsular polysaccharide production improved by 40 to 140%, compared to the wild type, with respect to the final product titer. A DO-stat fed-batch process on the 2L scale was also developed for the best performing integrative strain, EcK4r3, yielding 5.3 g∙L^-1^ of K4 polysaccharide. The effect of *rfaH* overexpression in EcK4r3 affected the production of lipopolysaccharide and the expression of genes involved in the polysaccharide biosynthesis pathway (*kfoC* and *kfoA*), as expected. An alteration of cellular metabolism was revealed by changes of intracellular pools of UDP-sugars which are used as precursors for polysaccharide biosynthesis.

**Conclusions:**

The present study describes the identification of a gene target and the application of a successful metabolic engineering strategy to the unconventional host *E. coli* K4 demonstrating the feasibility of using the recombinant strain as stable cell factory for further process implementations.

## Introduction

Chondroitin sulfate (CS) is a natural linear polysaccharide formed by disaccharide units of N-acetyl-D-galactosamine (GalNAc) and Glucuronic acid (GlcA) β 1:4 and β 1:3 linked. It is a ubiquitous component of the extracellular matrix of vertebrates where it exerts its established chondroprotective properties by improving the biosynthesis of connective tissue components and increasing the viscosity of synovial fluid at disease sites [1,2]. CS also inhibits cartilage degradative enzymes therefore it is widely used for the treatment of osteoarthrithis. Moreover, recent novel studies suggest its potential applicability for cancer prevention, formulation of skin substitutes, and vaccine development [3-5].

**Table 1:** **Summary of growth, K4 CPS production and relative yields obtained in shake flask experiments from the reference *E.coli *K4 and the recombinant strains**

**Strain**	**Carbon/Nitrogen**	**Biomass**	**μ**_**max**_	**K4**	**Y**_**K4/X**_	**Y**_**K4**/**S**_
		**(g**_**cdw**_∙**L**^**-1**^**)**	**(h**^**-1**^**)**	**(mg**∙**L**^**-1**^**)**	**(mg**∙**g**_**cdw**_^**-1**^**)**	**(mg**∙**g**^**-1**^**)**
*E.coli* K4	Gly/Soy	2.24	0.88	124	55	11
*E.coli* K4-pTrc*rfaH*	Gly/Soy	2.08	0.67	294	140	-
EcK4r1	Gly/Soy	2.49	0.76	174	70	-
EcK4r3	Gly/Soy	2.39	0.77	212	88	20
*E.coli* K4	Glu/Ye	2.89	1.03	190	66	18
EcK4r3	Glu/Ye	2.83	1.05	283	100	27

The concentration of capsular polysaccharide was measured in the broth at the end of the experiment after 24 h of growth. Y_K4/X_ indicates mg of K4 CPS produced per g of dry cells. Y_K4/S_ indicates mg of K4 CPS produced per g of carbon source consumed. μmax indicates the maximum specific growth rate in h^-1^. The values are averages of at least 4 separate experiments with a standard deviation below 10%. Abbreviations: Glu (Glucose), Gly (Glycerol), Ye (Yeast extract).

**Table 2:** Hydrolysis of the K4 CPS produced during shake flask experiments

	**pH=5**		**pH=6**	
**Time**	**K4**	**Defructosylated**	**K4**	**Defructosylated**
**(h)**	**(**mg∙L^-1^**)**	**(**mg∙L^-1^**)**	**(**mg∙L^-1^**)**	**(**mg∙L^-1^**)**
**3**	189	27	199	17
**6**	181	35	198	18
**16**	162	54	192	24

The polysaccharide (216 mg∙L^-1^) was incubated in the standard growth medium at pH=5 and pH=6 without cells for 16h. The temperature was kept constant at 37°C and agitation was set to 200 rpm. Samples were collected after 3, 6 and 16h of incubation to analyse defructosylation over time.

**Figure 1:**
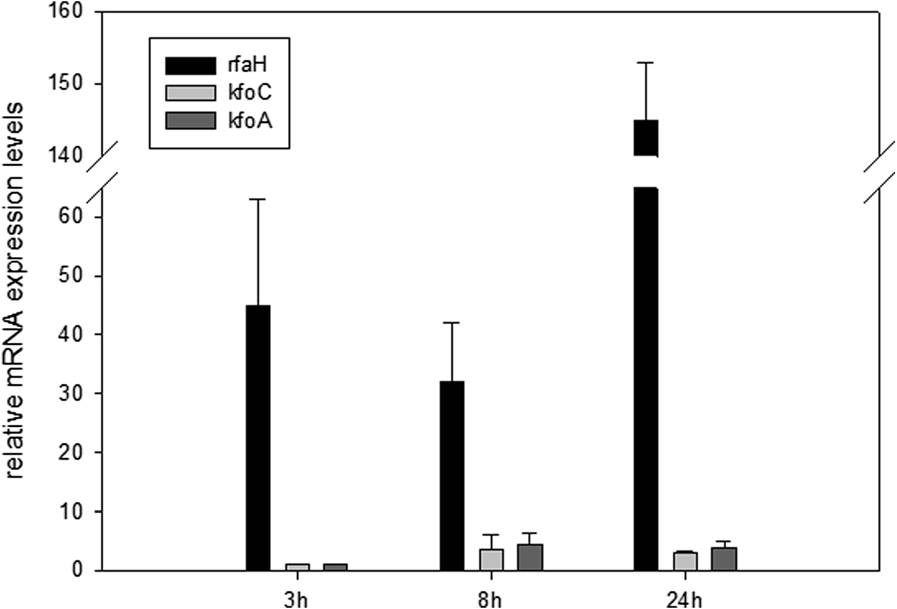
**Analysis of *rfaH*, *kfoC *and *kfoA *gene expression in *E.coli *K4 and EcK4r3 in shake flask experiments.** Histogram representing the fold overexpression of the genes under investigation in the recombinant strain EcK4r3 compared to the wild type *E.coli* K4.

**Figure 2:**
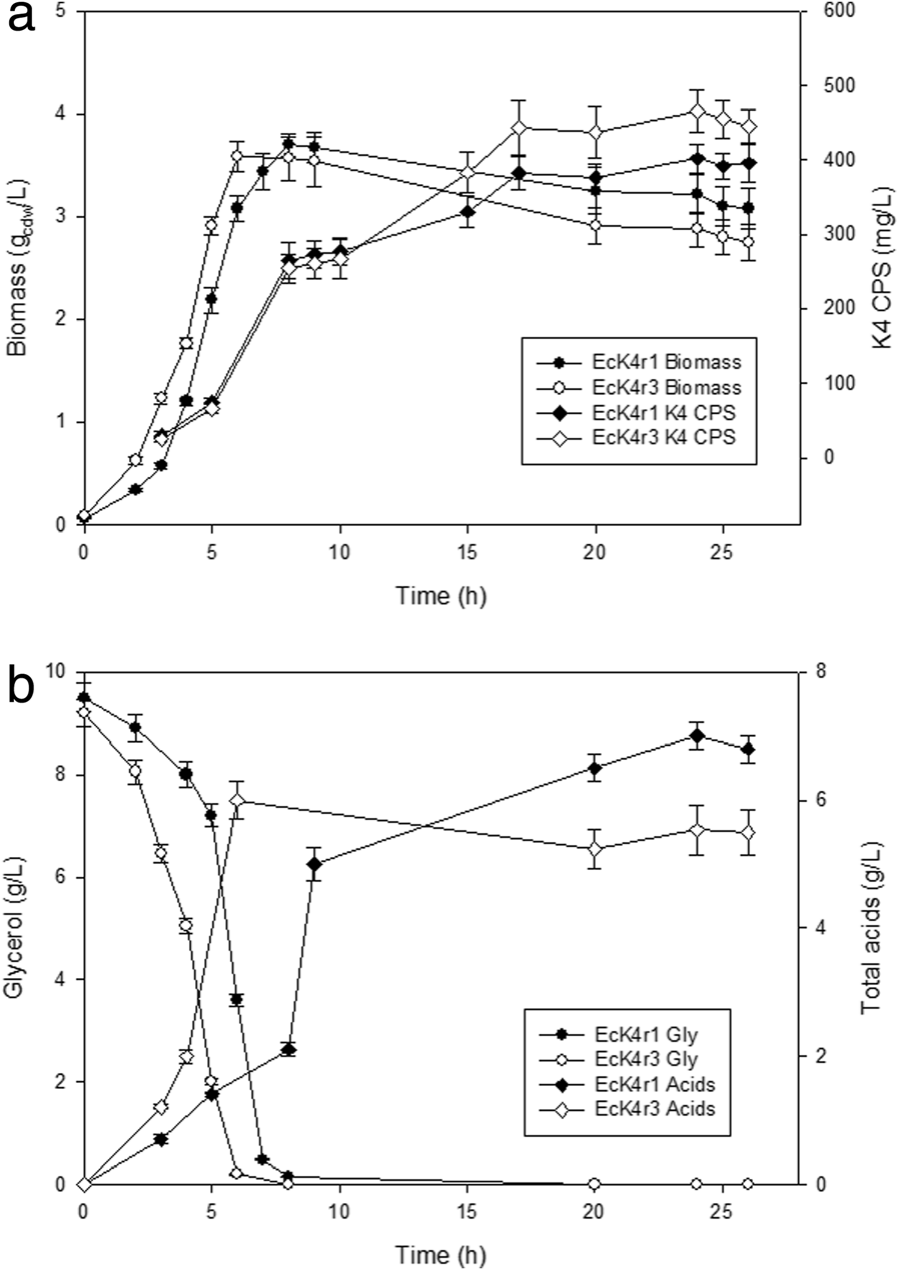
**Time course of 24h batch fermentations on the control medium: comparison of strains EcK4r1 and EcK4r3.** (**a**) Production of biomass and K4 CPS (**b**) Consumption of glycerol and production of total acids throughout the process.

**Table 3:** **Intracellular concentrations of UDP-Glu, UDP-GlcA and UDP-GalNAc in *E.coli *K4 (wt) and EcK4r3 during batch experiments**

		***E.coli *K4 (wt)**	**EcK4r3**
		**μmol∙g**_**cdw**_^**-1**^	**μmol∙g**_**cdw**_^**-1**^
5h	UDP-Glu	0.56	11.3
	UDP-GlcA	0.42	0.11
	UDP-GalNAc	3.77	1.63
8h	UDP-Glu	1.92	5.64
	UDP-GlcA	1.33	0.82
	UDP-GalNAc	0.61	0.51
24h	UDP-Glu	1.96	0.37
	UDP-GlcA	1.85	1.93
	UDP-GalNAc	0.07	0.05

Samples were collected after 5, 8 and 24h of growth. Experiments were performed in duplicate and the standard deviation was ≤ 15%.

The external coating, defined as capsule, of *Escherichia coli* K4 is composed of fructosylated chondroitin and therefore represents an unlimited source of precursor supply for the production of CS. Moreover the use of microbial polymer eliminates the risk of interspecies viral and prionic contaminations.

Several thriving research efforts regarded the set up of fermentation strategies targeting the maximization of capsular polysaccharide (CPS) production from *E. coli* K4 followed by recovery of a 90% pure polysaccharide and final chemical sulfation resulting in a polymer that is equivalent to the extractive counterpart [6-8]. Productivity was further increased by overexpressing the enzyme responsible for polymer assembly in the wild type (wt) strain using an inducible plasmid as expression system. However, although a 100% improvement in the final CPS titer was achieved, these experiments also revealed a great genetic instability of the recombinant strain, that limited process scale-up [9].

The K4 CPS belongs to group II K antigens. Capsules produced from *E. coli* K5 and K1 are well studied members of this group [10]. The structure of the cluster responsible for CPS biosynthesis is organised into three regions. Regions 1 and 3 contain the *kps* genes common to all group II members and mainly involved in the transport of the polymer in the periplasm and outside the cell wall [11,12]. The genes coding for enzymes that direct the synthesis and assembly of the final polysaccharide are found in region 2, that is the serotype specific. Besides the IS2 insertion sequence, region 2 in *E. coli* K4 comprises three genes of unknown function (*kfoB*, *kfoG*, *kfoD*), *kfoE* responsible for the addition of fructose residues [13]*, kfoA* coding for a UDP-glucose 4-epimerase, *kfoF* coding for a UDP-glucose dehydrogenase and the well characterised *kfoC* gene coding for chondroitin polymerase [14,15].

One of the factors regulating capsule expression is the transcriptional activator RfaH that carries out an antitermination process, that also requires the *cis*-acting operon polarity suppressor element (ops) located just upstream of a JUMP start (just upstream of many polysaccharide gene starts). The role of the ops element is to recruit RfaH, and therefore its presence on the nascent transcript is crucial. Deletion of the ops element results in fact in the lack of capsule expression in *E. coli* K5 [16]. The JUMP start sequence is also required for RfaH to function; indeed it is conserved and present at the 5′ end of RfaH-regulated operons, and it might be necessary to fold the mRNA into stem-loop structures that mediate these processes [17]. RfaH plays several roles in *E. coli* affecting the biosynthesis of lipopolysaccharide (LPS), O-antigen, and haemolysin, and the production of the F-factor sex pilus [17]. Its function is to control promoter distal gene expression by preventing the termination of transcripts and promoting transcription over long distances. In *E. coli* K5 RfaH seems to prevent the termination of region 3 transcripts allowing read through transcription, and thereby also regulating the transcription of region 2 [16]. *E. coli* K5 strains with mutations in the *rfaH* gene showed undetectable levels of expression of region 2 genes, and of the corresponding encoded proteins. The regulation of transcription of group II capsule gene cluster is quite complex, also depending on the activity of other regulator proteins namely H-NS (nucleoid associated protein) and Bip-A (tyrosine phosphorilated GTPase) that are involved in the thermal regulation of region 1 and 3 promoters [18].

In the present study an integrative expression cassette comprising the *rfaH* gene under the control of part of the constitutive promoter of the *gapA* gene, coding for glyceraldehyde 3-phosphate dehydrogenase, was constructed and introduced into wild type *E. coli* K4. The recombinant strains overexpressing the regulator demonstrated a significant increase of the CPS yield and proved suitable for the scale up of the fermentation process.

## Results

### Strain construction

The host *E. coli* K4 was modified by the addition of extra, plasmidic or integrative, copies of the *rfaH* gene. *E. coli* K4-pTrc*rfaH* possesses additional inducible copies of *rfaH* on the pTrc plasmid, under the control of the hybrid promoter consisting of the -10 region from *lacUV5* and the -35 region from *trpB*. Protein profiles of this strain grown in shake flask showed an overexpressed band of about 18 KDa by SDS-Page 3h after IPTG induction and the intensity of this band slightly increased after 8 and 24h of growth (data not shown). The sequence of the *rfaH* PCR product cloned in the pTrc plasmid was compared to that of *E. coli* K12 and silent point mutations, three transitions and three transversions, were identified (Additional file 1).

EcK4r1 and EcK4r3 were generated by the integration of a fragment containing the *rfaH* gene under the control of the P1 portion of the *gapA* promoter, targeting the endogenous plasmid pK4EC05 and the *lacZ* gene, respectively. The expression cassette also comprised the kanamycin resistance gene flanked by FRT sites that allowed the removal of the selection marker by means of a consecutive pop-out step, resulting in resistance-free strains.

### Shake flask experiments

Direct comparison of the three recombinant strains was performed in shake flask conditions on the control medium [19] to investigate physiological differences resulting from (i) an increased copy number of the *rfaH* gene (ii) the different expression systems used, and (iii) integration targeted to endogenous plasmid or genome (strains EcK4r1 and EcK4r3, respectively). All recombinant strains demonstrated a significant increase of CPS production after 24h of growth together with a higher Y_K4/X_, thereby clearly demonstrating the positive effect of a higher availability of RfaH on CPS production (Table 1). *E. coli* K4-pTrc*rfaH* was used in this study only as a tool to evaluate whether *rfaH* overexpression could boost CPS production, and after 24h of growth a 2.4-fold higher concentration of polysaccharide was in fact obtained with a 155% increase of the Y_K4/X_; a slightly lower growth rate and biomass production compared to the wild type strain was probably due to the higher metabolic burden.

The performance of integrative recombinants EcK4r1 vs. EcK4r3 was quite different in terms of final total polysaccharide produced and yield over biomass. As shown in Table 1 the percentage of increase of both polymer and Y_K4/X_ at the end of the growth, compared to the wild type strain, was almost two fold higher for EcK4r1 versus EcK4r3.

When testing *E. coli* K4 and EcK4r3 on a new medium containing glucose and yeast extract, an increase of biomass and K4 CPS was observed. Furthermore, also the yield of K4 CPS per gram biomass increased on the new medium for both strains, indicating a decoupling of polysaccharide and biomass formation (Table 1). EcK4r3 cultivations also showed the decrease of production of fructosylated biopolymer from 14 to 1% and the drastic reduction of the pH_24h_ from 6.4±0.28 (on the standard medium) to 5.2±0.14. In order to verify that the additional copies of *rfaH* did not modify the aggressiveness of EcK4r3, a MIC test was performed. In particular the antimicrobial resistance of EcKr3 was compared to that of the wild type strain by using a commercial kit. Results did not highlight any difference between the two strains.

### Hydrolysis of the K4 CPS

A partial defructosylation of the K4 CPS was obtained after the incubation at pH=5 and pH=6 in conditions simulating growth in shake flasks but without cells (Table 2). In particular at pH=5 the percentage of defructosylated polymer increased from about 12.5% after 3h of incubation to 25% at the end of the process. Defructosylation was less pronounced when incubating the polymer at pH=6.

### Analysis of gene expression

Quantitative real time PCR was used to analyse the level of expression of the *rfaH* gene in EcK4r3 grown on the medium containing glucose and yeast extract to investigate its expression levels and establish whether the presence of additional gene copies also affected RNA expression profiles of the *kfoA* and *kfoC* genes. Samples from shake flasks were collected during early exponential and mid to end stationary phases. Expression was normalised on the 16S rRNA. The overexpression folds are shown in Figure 1. The expression of all genes increased during growth in EcK4r3 compared to the wild type but, as expected, the increment of *rfaH* mRNA pools was higher compared to that of *kfoC* and *kfoA* transcripts.

### Batch experiments in 2L bioreactors

Twenty-six hours batch growths were conducted to analyse the physiology of strain *E. coli* K4-pTrc*rfaH,* however, results were completely opposite from those expected after shake flask experiments. The amount of K4 CPS produced was on average 190 mg·L^-1^, almost 40% lower compared to that obtained from cultivating the reference strain in the same conditions. A growth defect and the loss of recombinant plasmid already after the first 8h of growth were observed (data not shown), explaining the low Y_K4._

Batch experiments comparing strains EcK4r1 and EcK4r3 on the control medium are shown in Figure 2(a-b). The final titer of polysaccharide in the supernatant was higher by 34 and 55% compared to results obtained with *E. coli* K4 in the same growth conditions. EcK4r1 showed a lower growth, glycerol consumption and acid production rate (Figure 2b) compared to EcK4r3 and the concentration of total acids accumulated by the end of the process was higher. CPS production levels reached 402±20 and 466±25 mg·L^-1^,for EcK4r1 and EcK4r3, respectively.

The behaviour of EcK4r3 in the medium containing glucose and yeast extract was also analysed. Compared to the control medium only a slightly higher growth and carbon source consumption rate were observed, whereas the yield of K4 on biomass was unaffected. After 6h of growth the carbon source was exhausted, 4.2 g_cdw_·L^-1^ of biomass and 260 mg·L^-1^of K4 CPS were produced; the latter however doubled by the end of the process (24h) determining a final concentration of 532±30 mg·L^-1^of polysaccharide. The concentration of cell-bound and released LPS after 24h of growth was equal to 0.005 and 0.16 g·g_cdw_^-1^, respectively. Comparing the data to that obtained from the wild type strain a 1.58 and 1.55-fold increase were obtained for the two LPS fractions demonstrating the effect of *rfaH* overexpression on LPS production.

A hot methanol extraction method was used to determine the UDP-sugar precursors' content in fermentation samples by capillary electrophoresis. In particular biomass samples for the wt *E. coli* K4 (fermentation data not reported) and recombinant strain EcK4r3 after 5, 8 and 24h of growth were collected and analysed to identify eventual differences in UDP pools (Table 3). UDP-GlcA increased over time in both strains reaching after 24h a quite similar concentration of μmoles per g_cdw_; conversely, a 3.8 and 1.6-fold higher amount of sugar nucleotide is present in the wt after 5 and 8h of growth, respectively. The opposite trend is observed for UDP-GalNAc that decreases in the recombinant and wt strains; also in this case the precursors’s pool seems to be richer in the wt strain (2.3-fold higher) after 5 h of growth whereas more similar concentrations in the two strains are noticed after 8 and 24 h. Finally, *E. coli* K4 and EcK4r3 greatly differ for the content of intracellular UDP-Glucose (UDP-Glu) that shows opposite growing trends in the two strains, and quite diverse concentrations (Table 3).

### DO-Stat fed-batch experiment

**Figure 3:**
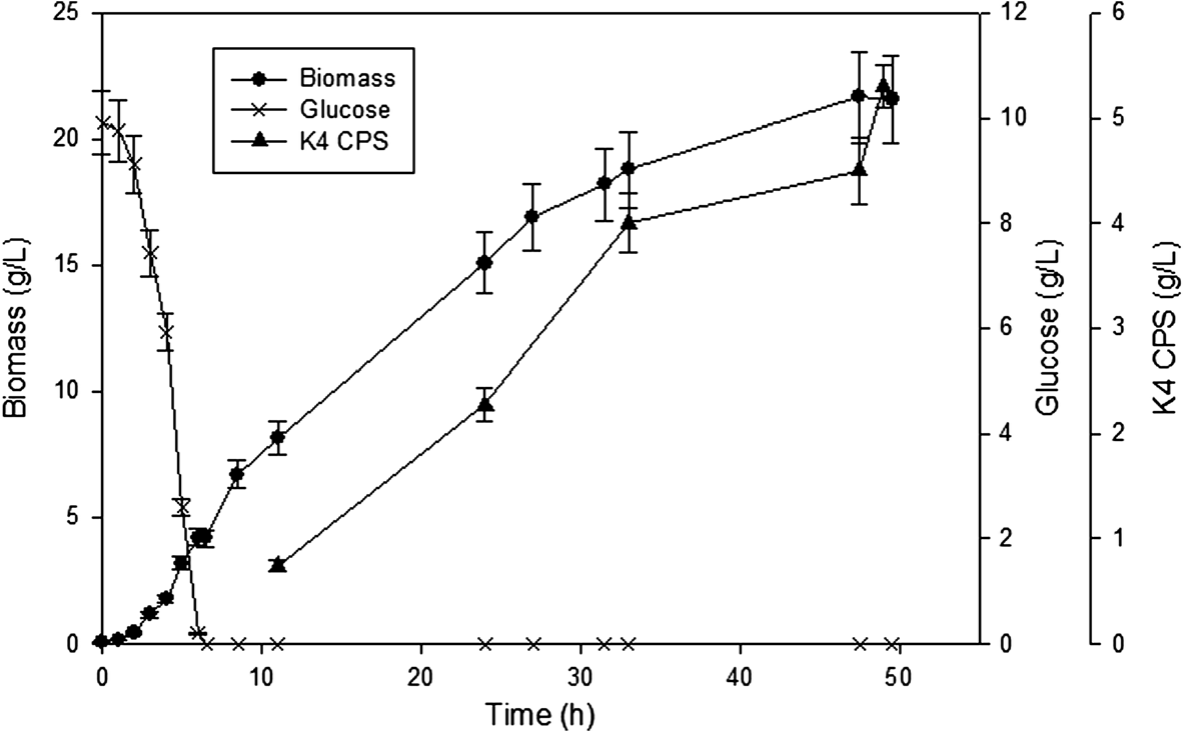
**Production of K4 CPS in 2L DO-Stat fed-batch experiments.** Time course of biomass, polysaccharide production and residual glucose.

EcK4r3 was analysed in fed-batch fermentations using a DO-stat feeding strategy in order to meet the strain’s metabolic requests thus keeping overflow metabolism under control. On average within 49.5h of process the cell density reached 21.7±1.7 g_cdw_∙L^-1^ and 5.1±0.2 g∙L^-1^ of K4 CPS were attained (Figure 3). Compared to batch experiments on the same medium the Y_K4/X_ increased from 0.13±0.01 to 0.23±0.01 g_CPS_∙g_cdw_^-1^ whereas the Y_X/S_ decreased from 0.42±0.03 to 0.164±0.026 g_cdw_∙g_S_^-1^.

## Discussion

Several microbial strains representing natural sources of GAG and GAG-similar molecules have so far been isolated and a great progress concerning the improvement of yields through the optimization of fermentation strategies was recently described [6,20]. Besides classical process optimization also genetic engineering of the strain is crucial for improving the efficiency of natural hosts. The so far reported metabolic re-tooling strategies mainly focused on increasing the supply of sugar precursors and of enzymes responsible for chain polymerization [6].

The transcription of region 2 of the CPS cluster of group II *E. coli*, that includes *E. coli* K4, is driven by a promoter located just downstream of region 3, consequently region 2 and 3 genes are transcribed as a single mRNA unit. Expression of group II K antigens requires the presence of the RfaH factor having the role of antiterminator thus providing transcription of promoter distal genes [21]. Previous work has indicated that loss of the RfaH protein in group II *E. coli* results in the decreased expression of region 2 genes [16] and K capsule [22]. It was therefore interesting to investigate whether, conversely, protein overexpression could lead to an increase in capsular polysaccharide production. At first the recombinant plasmid pTrc-*rfaH* containing the *rfaH* gene under the control of the Trc inducible promoter was introduced into *E. coli* K4 cells. Cells harbouring the above mentioned construct produced a protein of the expected molecular weight that increased over time in shake flask experiments and a significant increase in polysaccharide production was obtained after 8h and 24h of growth. The *E. coli* K4-pTrc*rfaH* recombinant strain only served as a proof of principle to determine whether the increased availability of RfaH would trigger CPS production. In fact, as already found previously for a similar expression construct [9], also in this case a severe loss of recombinant plasmid was observed in fermentation conditions thereby harshly affecting the productivity of the process. This finding therefore confirms that conventional plasmid driven expression constructs are suitable for investigating possible metabolic engineering targets in shake flasks but do not allow the scale up of the process with *E. coli* K4.

An expression cassette containing the *rfaH* gene under control of the P1 partial constitutive promoter of the glyceraldehyde-3-P dehydrogenase was designed and used to obtain integrative strains. As previously demonstrated *E. coli* K4 possesses an endogenous mobile element indicated as pK4EC05 that is stably inherited and shows homology to large conjugative plasmids [9]. pK4EC05 and the genome were used as integration targets for the construction of strains EcK4r1 and EcK4r3, respectively, to investigate whether the integration site could have an impact on improving CPS production. Results clearly show that growth of EcK4r1 and EcK4r3, and biomass formation were not affected by the introduction of recombinant DNA. Both strains demonstrated a significantly higher amount of polysaccharide in shake flask experiments on the control medium compared to the wt. A better performance was obtained from EcK4r3 that almost reached the titer produced from *E. coli* K4-pTrc*rfaH*. One major goal of this study was to investigate the stability of the newly constructed recombinant strains, a key feature in the attainment of master and working cell banks for biopharmaceutical applications. When shifting from shake flask to fermenter experiments the Y_K4/X_ for the wild type strain improves by 50%, specifically from 55 to 82mg_CPS_·g_cdw_^-1^[19]. The same Y_K4/X_ percentage increase was obtained when scaling up the batch process for EcK4r1 and EcK4r3. Also the Y_K4/X_ percentage increment for the two recombinant strains observed in shake flask experiments, in comparison to the wild type, was maintained in fermenter experiments (60% for strain EcK4r3 and 30% for strain EcK4r1). This conserved behaviour under the two different cultivation conditions is clearly demonstrating strain stability and overcomes the previously described problems. The development of biotech processes is often hindered by the use of hosts harbouring recombinant plasmids that can trigger stress responses that finally limit biomass and product yield, as observed for *E. coli* K4-pTrc*rfaH*. Similar restraints were not encountered for strain EcK4r1; as a matter of fact we observed that this endogenous element can host foreign DNA and keep it over generations and time, confirming its key importance for strain survival and it’s suitability for targeted gene integrations.

Fermentation experiments on the control medium in which the physiology of the integrative strains was studied confirm the superior performance of EcK4r3. Data highlighted a slightly faster growth for the latter that is reflected into the steeper substrate consumption and corresponding acid production rates. The significantly higher concentration of total acids (>25%) produced by EcK4r1 suggests a different carbon distribution which might influence the entire CPS biosynthesis process, resulting in a lower concentration of polymer. However, a key role might also be played by the number of gene copies integrated in the recombinant strains and by the integration site itself.

**Figure 4:**
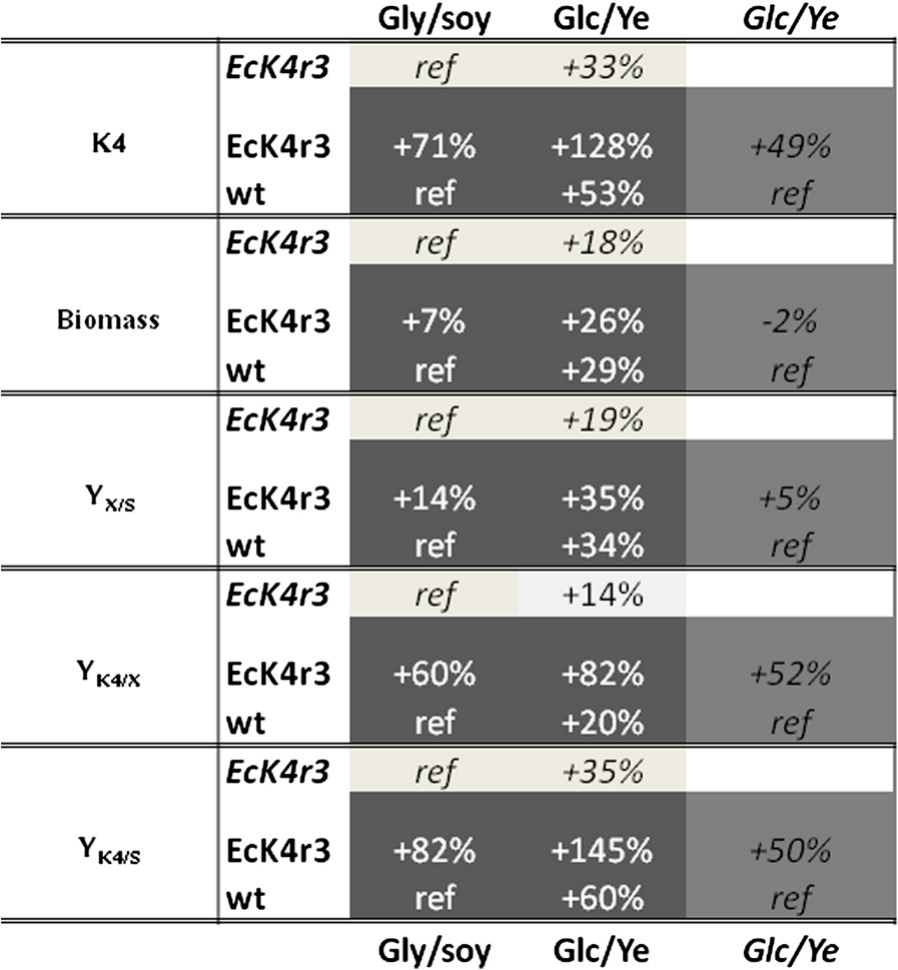
**Relative changes of K4 CPS, biomass, and yields on different media for EcK4r3 and *E.coli *K4 (wt).** Percentages in boxes with the same grey tone are referred to the combination of strain and medium indicated with ref (equal to 100%). Abbreviations: Glu (Glucose), Gly (Glycerol), Ye (Yeast extract).

The use of glycerol as main carbon source in industrial microbiology is advantageous due to its low cost and handling ease. Nevertheless glucose is often preferable in large scale fed-batch fermentation processes. In this perspective an alternative medium containing glucose as main carbon source was evaluated. Also soy peptone, the main nitrogen source, was replaced by yeast extract that better supported growth and CPS production. A comparison of growth and CPS production for EcK4r3, the best performing integrative strain, and the wild type, on the two media under analysis is schematically represented in Figure 4. Considering all parameters studied best performances for both strains were obtained in the medium containing glucose and yeast extract. However, interestingly, the potential of the recombinant strain seems to be more expressed on the control medium since the improvement of all yields is higher, maybe indicating the occurrence of a certain type of limitation. Moreover the increment of biomass, K4 CPS, and relative yields observed for the wt strain following medium substitution, is lower for EcK4r3 further supporting the previous hypothesis. This may indicate that CPS production is subjected to various control levels one of which is RfaH-dependent, whereas the others controlled by medium components. Nevertheless, EcK4r3 demonstrated a yield of polysaccharide (mg·(g_cdw_·g_subS_)^-1^) in batch experiments on this medium never described before, that is similar to that obtained with hyaluronic acid-producing *Streptococcus* strains [23,24] already used for industrial manufacturing processes. Compared to growth on the standard medium containing glycerol as the main carbon source we observed both for the wild type and for EcK4r3 an increase in the production of defructosylated polymer when glucose and yeast extract are present in the medium. Growth of both strains on the standard medium generates a final pH of about 6.4 whereas on the richer medium due to the higher amount of acids produces the pH is around 5. Chemical hydrolysis experiments of the K4 CPS in conditions simulating growth for 16h at pH 5 and 6 demonstrated a partial hydrolysis of the polysaccharide that is about 2-fold more pronounced at the lower pH. We therefore suggest that this might be one of the causes responsible for the presence of a polymer that is mostly defructosylated. However, probably several concurrent phenomena contribute to the production of mainly defructosylated polymer. For example since the growth and carbon consumption rates of both strains are higher on glucose another cause may be attributed to kinetic differences between polymerization and fructosylation and also to the channelling of fructose towards the synthesis of GalNAc that may also contribute to explaining the data observed.

To further improve the concentration of polysaccharide produced, a fed-batch process using a DO-stat feeding approach was developed. This technique allowed to satisfy the strain’s metabolic demand avoiding cell starvation or the accumulation of excess carbon thereby reaching the highest titer of K4 CPS produced through fermentation experiments up to date (5.3 g∙L^-1^). Compared to batch experiments on the same starting medium the amount of K4 CPS produced by EcK4r3 increased by 10-fold. Moreover growth conditions in fed-batch/DO-stat mode seemed to promote polysaccharide production to the detriment of biomass, since a 1.8-fold higher Y_K4/X_ was observed whereas the Y_X/S_ decreased by 2.6-fold compared to batch experiments.

Previous fermentation experiments on the wild type *E. coli* K4 performed by our group showed a significant improvement of the final K4 CPS concentration by using a membrane bioreactor combined with a fermentation strategy based on in situ product removal [25]. ECK4r3 would allow the use of well scalable fed-batch processes that do not require additional devices inside the fermentation vessel, which would be a step towards the actual production-scale of tons per year of chondroitin sulfate.

In order to investigate the increased availability of the antiterminator at the transcriptional level, the expression of *rfaH* in EcK4r3 was investigated through qPCR to analyse the impact of the additional p*gapA* regulated copy/ies on its expression. In particular the P1 site of the multipromoter system was used in this study [26]. As expected a significative increase of the *rfaH* mRNA pool was observed throughout growth with a peak after 24h. Stevens and coworkers [16] demonstrated that RfaH does not directly increase the initiation of region 2 transcripts whereas it supports their activity to obtain adequate levels of expression of the genes that are required to synthesize the K5 capsular polysaccharide. We analysed the expression of two genes belonging to region 2, namely *kfoC* and *kfoA*. Both genes were positively affected by *rfaH* overexpression without significative differences among the two transcripts. RfaH regulates bacterial operons engaged in the production of also other extracellular components involved in the virulence of pathogens such as the LPS, α-haemolysin toxin and F pilus. In fact, deletion of *rfaH* abolished the virulence of uropathogenic *E. coli* due to downregulation of several virulence factors [22]. To further deepen the study, production of cell-bound and released LPS in EcK4r3 was also investigated. The same CPS to LPS ratio observed for the wild type strain (2:1) is conserved in EcK4r3, although the latter demonstrates a 1.5/1.6-fold increase of both the cell-bound and released LPS portions, in accordance with the increment of K4 CPS production. These results clearly demonstrate the broader effect of *rfaH* overexpression.

A higher production of capsular polysaccharide, as in EcK4r3, does not necessarily imply a variation in the concentration of intracellular pools of precursor UDP-sugars; the former could in fact be due to a higher flux through the pool. However, it was interesting to find that *rfaH* overexpression affected both concentration values and time course profiles of UDP-Glu, UDP-GalNAc and UPD-GlcA, analysed in this work. The major difference, compared to the wt strain, regarded UDP-Glu. Wu and co-workers [27] cultivating *Lactobacillus casei* found an increase in the abundance of enzymes responsible for the biosynthesis of UDP-Glu and of precursor sugar-nucleotides for the synthesis of exopolysaccharides, in the early stationary phase. A similar trend was observed for the wt *E. coli* K4, whereas in EcK4r3 not only the intracellular concentration of UDP-Glu decreased towards the end of growth, but a 20-fold higher concentration compared to the wt, was observed when growth slowed down (5h). *kfoF*, belongs to region 2 and codes for a UDP-glucose dehydrogenase that converts UDP-Glu into UDP-GlcA. The induction of the gene, due to *rfaH* overexpression, might have increased the need for UDP-Glu that is an essential intermediate also for other biosynthesis processes (eg. Biosynthesis of cell wall components), so that the metabolism responded by increasing the available pool of this substrate. In both strains UDP-GlcA and UDP-GalNAc exhibited the same trends over time, however a lower intracellular availability of both sugar-nucleotides, that is most pronounced after 5h of growth, was observed in EcK4r3 compared to the wild type which might mirror the higher polysaccharide production rate of the recombinant strain. Overall *rfaH* overexpression seems to affect the intracellular concentration of UDP-sugar precursors further supporting an extensive metabolic response at least regarding the pathways involved in polysaccharide biosynthesis.

## Conclusions

**Figure 5:**
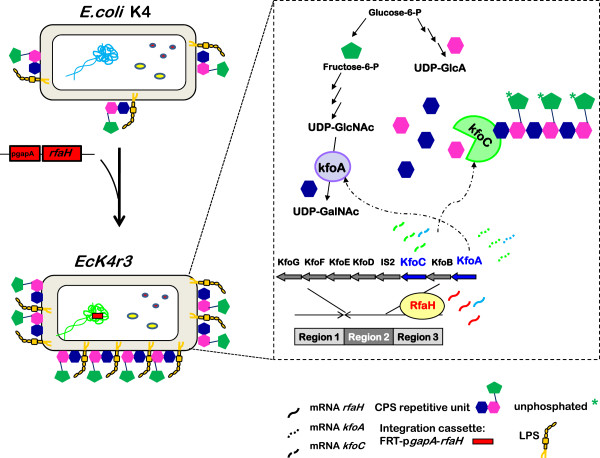
**General overview on the effect of inserting additional copies of *rfaH *in *E.coli *K4.** It shows that overexpression of *rfaH* leads to higher production of capsular polysaccharide that is probably due to an increase of expression of the genes belonging to region 2; in particular the mRNA levels of *kfoA* and *kfoC* were analysed in this study. The figure also indicates that *rfaH* overexpression increases lipopolysaccharide production.

Overall in the present work a new engineered chondroitin production host was constructed by introducing additional copies of the *rfaH* gene in the unconventional strain *E. coli* K4. Homologous overexpression of the gene not only improved CPS production in shake flasks but also increased the final titer of polysaccharide in fermenter experiments in batch and fed-batch mode, thereby proving the possibility of achieving stable integrations in this peculiar capsulated strain. Furthermore the construct developed in this work does not require induction by IPTG or selection pressure (e.g. antibiotics) during the cultivation which makes the foreseen production process cheaper and reduces the impact on the environment. This work also shows that the know how acquired with studies carried on the wild type, and the fermentation processes developed, can be easily transferred to genetically engineered strains simplifying the approach towards the set up of large scale biotech production processes. A global overview of the results obtained in the present work is depicted in Figure 5.

## Materials and Methods

### Medium

The standard medium used for all shake flask and fermenter production studies consisted of a defined salts medium (KH_2_PO_4_ 2 g∙L^-1^; K_2_HPO_4_ 9.7 g∙L^-1^; Na_3_C_6_H_5_O_7_ 0.5 g∙L^-1^; (NH_4_)_2_SO_4_ 1 g∙L^-1^; MgCl_2_ 0.1 g∙L^-1^) supplemented with glycerol or glucose (10 g∙L^-1^) as the main carbon source and neutralised soy peptone (1 g∙L^-1^) or yeast extract (2 g∙L^-1^), respectively, as additional nitrogen source (Oxoid). Luria-Bertani (LB) medium was used during transformation experiments. Transformed cells were grown on LB medium supplemented with ampicillin or kanamycin (50 μg∙mL^-1^). The wild type strain used in all experiments was *E. coli* K4 serotype O5:K4:H4 (CCUG 11307), purchased from the Culture Collection University of Göteborg.

### Materials

Genomic DNA, plasmid DNA, and RNA were isolated using Qiagen DNeasy kit, Qiagen miniprep kit, Qiagen RNeasy kit (Qiagen, Valencia, CA) respectively according to the manufacturer’s instructions. Restriction endonuclease digestions, DNA ligations, SDS-PAGE and agarose gel electrophoresis were performed using standard techniques [28].

### Construction of the *rfaH* overexpressing strains

Primers used for the construction of strains described in this study are reported in Table 4. Amplification of the *rfaH* gene and of the P1 *gapA* promoter for the construction of all strains were performed from *E. coli* K4 chromosomal DNA. Primers for the amplification of the gene and of the promoter were designed based on the sequence of *rfaH* and p*gapA* of *E. coli* K12 present in the database. Polymerase chain reaction (PCR) was carried out with Expand High fidelity PCR System (Roche, Monza, Italy) according to the manufacturer’s protocol. DNA fragments were recovered from agarose gels using the Qiaquick gel extraction kit (Qiagen, Valencia, CA). Restriction endonucleases were purchased from New England Biolabs and ligases were purchased from Invitrogen (Carlsband, CA). Nucleotide sequencing of all PCR fragments cloned was carried out at BMR Genomics (Padova, Italy) to verify that the sequences were correct.

**Table 4:** **Sequences of the primers used for constructing strains *E.coli *K4-pTrc *rfaH *, EcK4r1 and EcK4r3, and for real time PCR experiments**

***Primer***	**Sequence**
*rfaHpTrc1*	5′-TAAAGGAGGTATACAAAAATGCAATCCTGGTATTTACTGTAC-3′
*rfaHpTrc2*	5′-TTAGAGTTTGCGGAACTCGGTAT-3′
*16Sq_Up*	5′- GGTGTAGCGGTGAAATGCGTAGAG-3′
*16Sq_Dw*	5′- CAAGGGCACAACCTCCAAGTC-3′
*qkfoC_Up*	5′-TTTTCCCTGCCGCACGATCC-3′
*qkfoC_Dw*	5′-TTCGGTCTGTTGAAGGAGCAATGG-3′
*qkfoA_Up*	5′-GGATTTAGCGGAAGGGCATGTG-3′
*qkfoA_Dw*	5′-TTCTGGTGATGACCAACTTTCAGC-3′
*P1=pK4EC05_Up*	5′-CAGCACAGCAGAGCGAAGTGCATCATATCCTTCCAGATTTAAATTCTTCAAATTAACCCTCACTAAAGGGCG-3′
*P2=pk4EC05_Dw*	5′-AAACTGTGATCGGGCGTAGGAACCCGCGTAGTCATCGTCGGCGCAGAAGTTTAGAGTTTGCGGAACTCGGTAT-3′
*P3=LacZ_Up*	5′-CACCCTGGCGCCCAATACGCAAACCGCCTCTCCCCGCGCGTTGGCCGATTCATTAATGCAGCTGGCACGACAGGTTTCCCGACTGGAAAGCGGGCAGTGAAATTAACCCTCACTAAAGGGCGG-3′
*P4=LacZ_Dw*	5′- AAAAGAATAAACCGAACATCCAAAAGTTTGTGTTTTTTAAATAGTACATAATGGATTTCCTTACGCGAAATACGGGCAGACATGGCCTGCCCGGTTATTTAGAGTTTGCGGAACTCGGTATTC-3′
*P5=FRT_Dw*	5′-TAATACGACTCACTATAGGGCT-3′
*P6=pGapA_Up*	5′-CCCTCGAGGGCCTTTAAAATTCGGGGCGCCGA-3′
*P7=pGapA_Dw*	5′-CGGGATCCCGATATTCCACCAGCTATTTGTTAG-3′
*P8=rfaH_Up*	5′-CGGGATCCCGATGCAATCCTGGATTTTACTGTAC-3′

### Strain *E. coli* K4-pTrc*rfaH*

*rfaH* was TA-cloned into pTrcHis (Invitrogen) not in frame with the N-terminal his tag contained in the vector. The forward primer contained a stop codon and a new ribosome binding site. Primers used for the amplification are rfaHpTrc1 and rfaHpTrc2 (Table 4).

### Strain EcK4r1- EcK4r3

**Figure 6:**
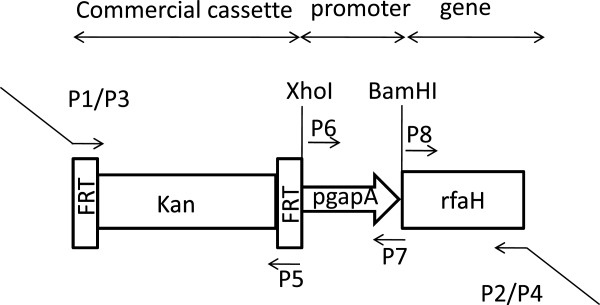
Schematic representation of the integration cassette used for the construction of EcK4r1 and EcK4r3.

The recombinant strains EcK4r1 and EcK4r3 were constructed using the Gene deletion kit (Gene Bridges). The provided integration cassette was modified by the addition of the *rfaH* gene under the control of the *gapA* promoter just downstream of the kanamycin gene (Figure 6). *E. coli* K4 wt chromosomal DNA was used as template for the amplification of the glyceraldehyde-3-phosphate dehydrogenase promoter and the *rfaH* gene. All primers used for strain construction are reported in Table 4. A scheme of the resulting cassette is depicted in Figure 6. The pK4EC05_Up and pK4EC05_Dw oligos contain a 50bp region similar to the 5' and 3′portion of the PK4EC05 plasmid, respectively, to target the recombination event for the construction of strain EcK4r1.

The cassette used for the achievement of strain EcK4r3 was amplified with primers LacZ_Up and LacZ_Dw containing 100bp region similar to the 5' and 3′portion of the *E. coli* K4 genome, respectively, to target the recombination event.

### Transformation

For the construction of all strains *E. coli* K4 electrocompetent cells were prepared according to the instrument instructions and transformed through electroporation using a Bio-Rad Gene Pulser (2mm cuvettes, 2.5 kV, 200 Ω, 25 μF). *E. coli* K4 was transformed with the vector pTrc-*rfaH* and plated on LB plates supplemented with 50 μg·mL^-1^ ampicillin to select for positive clones. Strains EcK4r1 and EcK4r3 were obtained following the instructions of the kit.

### Strain characterization

The Minimum Inhibitory Concentration (MIC) of antibiotics, antifungal and antimycobacterial agents was established by performing the Etest® (BioMerieux Italia S.p.a.). Etest consists of a predefined gradient of antibiotic concentrations on a plastic strip and it was used to compare *E. coli* K4 and EcK4r3. The analysis was performed by the group of Prof. Donnarumma at the Department of Microbiology, Second University of Naples.

### Gene expression

Quantitative real-time PCR was performed on *E. coli* K4 wild type and EcK4r3 recombinant strains to compare the mRNA levels of the genes of interest.

Three time points were chosen for analysis corresponding to 3, 8 and 24h of growth. RNA was treated with DNase using the DNA-free kit following the supplied protocol (Ambion Inc Austin, TX) and 500 ng were used for reverse transcription with the Reverse Transcription System (Promega, Madison, USA). PCR amplification was performed in an iQ5 instrument (Biorad California, USA). The 16S rRNA was used to normalise expression of *rfaH*, *kfoC* and *kfoA* genes. The primers used for the experiment are reported in Table 4.

Amplification was carried out in 25 μL containing 5 μL cDNA, 12.5 μL of iQ Syber Green Supermix (Biorad, California, USA) and 0.5 μL of each primer at a concentration of 0.4 μM. After incubation at 95°C for 3 min, amplification proceeded with 40 cycles of 95°C for 10 s and 62°C for 1 min. The efficiencies of the primer sets were measured using a dilution series of cDNA. The raw threshold cycle (Ct) values were converted to relative expression levels by the 2^(-ΔΔCt)^ method [29]. Three biological samples each in triplicate were analysed for every timepoint in the wild type and recombinant strains.

### Shake flask experiments

Shake flask experiments were performed in order to evaluate the effect of *rfaH* gene overexpression on the production of K4 polysaccharide. Before each experiment cells from 20% (*w/v*) glycerol stock preparations were streaked on agar plates and grown overnight (o/n) at 37°C. Single colonies were then used to inoculate pre-cultures that were incubated o/n at 37°C in shaking conditions.

The medium used for all shake flask production studies is described in the medium section. For strain *E. coli* K4-pTrc*rfaH* 100 μg∙L^-1^ ampicillin were supplemented before each experiment.

For all experiments 200 mL cultures of wild type and recombinant *E. coli* K4 were grown in 1 liter baffled flasks, keeping a 1:5 medium/air volume ratio, at 37°C and 200 rpm in a rotary shaker incubator (model Minitron, Infors, Bottmingen, Switzerland). For the strain *E. coli* K4-pTrc*rfaH* the cultures were grown until the OD_600_ reached 0.7 and at that point 0.4 or 1 mM β-isopropylthiogalactoside (IPTG) was added to the broth.

Strains EcK4r1 and EcK4r3 were grown for 24h without the addition of antibiotic and inducer. Samples were withdrawn during the course of the experiment to analyse polysaccharide production. Every shake flask experiment was repeated at least four times.

### Fermentation experiments

Fermentation experiments were carried out as biological triplicates in a Biostat CT reactor (Sartorius Stedim; Melsungen, Germany), 2 L working volume. A constant pH of 7.5 was maintained via automated addition of 30% v/v NH_4_OH and 30% v/v H_2_SO_4_.

Seed cultures were prepared by inoculating a single colony in 200 mL of medium in 1L baffled shake flasks. The flasks were incubated overnight at 37°C and 200 rpm. For the duration of all cultivations 5 mL samples were withdrawn from the reactors at regular time intervals for the determination of substrates, extracellular metabolites and polysaccharide quantification.

For batch experiments the concentration of dissolved oxygen (DO) was maintained above 20% varying the air flow rate (1–1.5 L∙min^-1^) and stirring rate (200–800 rpm) according to oxygen demand. The media used for all experiments were that described in the medium section. For the strain *E. coli* K4-pTrc*rfaH* 100 μg∙L^-1^ ampicillin were added to the medium before each experiment and the cultures were grown until the OD_600_ reached 0.7 and at that point 0.4 or 1 mM β-isopropylthiogalactoside (IPTG) was added to the broth.

For fed-batch experiments cells were grown in the semidefined medium containing glucose and yeast extract. A DO-Stat controlled feeding strategy with a pO_2_ set point equal to 35% of air saturation was used. After the batch phase, a concentrated nutrient solution (450 g∙L^-1^ glucose and 90 g∙L^-1^ yeast extract, inorganic salts 20-fold concentrated) was fed to the culture in the following 42.5h of growth.

### Extraction of the LPS

The biomass obtained from fermenter experiments was suspended in 90% v/v pre-warmed phenol. A volume of pre-warmed water was then added to the suspension and incubated for 30 minutes at 68°C in stirred conditions.

The solution was then centrifuged at 9000x*g* for 30 minutes at 4°C and the upper phase containing the LPS was extracted and preserved. This procedure was repeated for three times. The three fractions recovered were dialyzed against water (6000–8000 Da membranes) for two days. The upper phase was further treated for the quantification of the O-chain. Hydrolysis was performed adding 1% v/v acetic acid to the solution that was then incubated for 1.5 h at 100°C. The sample was then centrifuged at 9000xg, at 4°C for 30 minutes and the supernatant was analyzed through capillary electrophoresis as described in the following section (Quantification of CPS, LPS and UDP-sugars)

### UDP-sugar extraction

UDP-sugars were extracted from biomass samples by slightly modifying the method reported by Maharjan and Ferenci [30]. Briefly, broth samples corresponding to about 0.8 mg_cdw_ were cooled at 0°C in an ice-water bath and immediately centrifuged at 16000xg and 0°C for 5 minutes. Pellets were resuspended in 1mL of 50% v/v methanol and incubated at 70°C for 30 minutes. The supernatant was recovered by centrifugation and concentrated in a vacuum centrifuge to 70 μl for capillary electrophoresis analysis as described in the following section.

### Hydrolisis of the K4 CPS

The K4 CPS produced in shake flask cultivations was used for an experiment of chemical hydrolysis. The polysaccharide (216 mg∙L^-1^) was incubated in the standard growth medium at pH = 5 and pH = 6 for 16 h. The temperature was kept constant at 37°C and agitation was set to 200 rpm. Samples were collected after 3, 6 and 16h of incubation and analysed by capillary electrophoresis.

### Quantification of CPS, LPS and UDP-sugars

Broth samples were collected from shake flask and fermentation experiments and centrifuged at 1700xg. Supernatants were then ultrafiltered on 10 KDa centrifugal filter devices (YM-10 Centricon, Millipore, Bedford, MA, USA) at 5000xg and concentrated to 1/10th of their initial volume. The retentate was twice diafiltered and then analyzed for the content of K4 polysaccharide and LPS.

Capillary electrophoresis analysis were performed on a Beckman-Coulter HPCE instrument (P/ACE MDQ, Palo Alto, CA, USA) equipped with a diode array detector and a UV lamp, using an uncoated fused-silica tube (70 cm of total length, 60 cm of effective length, 50 μm I.D.) at 25°C. Separation and quantification of the K4 and K4 defructosylated polysaccharides and of the O-chain and LPS were performed according to the previously described method [31]. The same equipment was used for the determination of UDP-sugar concentrations by slightly modifying the method reported by Xu and colleagues [32]. In particular a buffer containing sodium phosphate 50mM, SDS 60mM and sodium borate 20mM, pH 9 was used for analysing the samples. Runs were performed at 18°C and 22 kV. UDP-Glu, UDP-GalNAc and UDP-GlcA were detected at λ = 200 nm. The concentration of standards used for the construction of calibration curves ranged between 0.013 to 1.7 mM for UDP-Glu and UDP-GalNAc, and from 0.026 to 1.7 mM for UDP-GlcA. Due to the complexity of the samples co-elutions with the pure standards were also performed to ascertain the identity of the different precursors.

## Abbreviations

CPS: Capsular polysaccharide; DO: Dissolved oxygen; GAGs: Glycosaminoglycans; CS: Chondroitin sulfate; HA: Hyaluronic acid; wt: Wild type

## Competing interests

The authors declare no competing interests.

## Authors’ contribution

DC wrote the manuscript; DC, EC and AR performed experiments; DC, EC, MDR, AR and CS analysed the data; DC and CS conceived the work; all authors read and approved the manuscript.

## Supplementary Material

Additional file 1 **Sequence of rfaH in *E. coli *K4.** Click here for file
