# Supplementary material for: Homologous overexpression of rfaH in E. coli K4 improves the production of chondroitin-like capsular polysaccharide
Source: Microb Cell Fact. 2013 May 9;12:46. doi: 10.1186/1475-2859-12-46 (PMC3673904; doi:10.1186/1475-2859-12-46)
Supplement: Additional file 1 — Sequence of rfaH in E. coli K4. [file 1475-2859-12-46-S1.doc]

ATGCAATCCTGGTATTTACTGTACTGCAAGCGCGGGCAACTTCAACGTGCCCAGGAACACCTCGAAAGACAGGCTGTGAATTGCCTGGCACCGATGATCACCCTGGAAAAAATCGTGCGTGGAAAACGTACTGCAGTCAGTGAGCCATTGTTTCCCAACTACCTGTTTGTCGAATTTGATCCAGAAGTGATTCATACCACGACTATCAACGCGACCCGCGGTGTCAGTCACTTCGTGCGCTTTGGCGCGTCGCCAGCGATAGTCCCATCGGCGGTGATTCATCAGCTATCGGTATATAAACCGAAAGACATTGTCGATCCGGCAACCCCTTATCCGGGAGATAAGGTGATTATTACCGAAGGCGCGTTCGAAGGCTTTCAGGCCATTTTCACCGAACCCGATGGTGAGGCTCGCTCCATGCTATTGCTTAATCTTATTAATAAAGAGATTAAGCACAGTGTGAAGAATACCGAGTTCCGCAAACTCTAA
